# The effect of selective serotonin reuptake inhibitors on cognitive function in patients with Alzheimer’s disease and vascular dementia: focusing on fluoxetine with long follow-up periods

**DOI:** 10.1038/s41392-019-0064-7

**Published:** 2019-08-30

**Authors:** Yi Xie, Pei-Pei Liu, Ya-Jun Lian, Hong-Bo Liu, Jian-Sheng Kang

**Affiliations:** 1 Clinical Systems Biology Laboratories, The First Affiliated Hospital of Zhengzhou University, Zhengzhou, 450052 Henan China; 2 Department of Neurology, The First Affiliated Hospital of Zhengzhou University, Zhengzhou, 450052 Henan China

**Keywords:** Neuroscience, Neurological disorders

Dear Editors,

Alzheimer’s disease (AD) and vascular dementia (VaD) are two of the most common forms of dementia, resulting in increased disability and mortality, impaired quality of life, and serious burdens on society and caregivers. However, the presently available drugs can only alleviate symptoms for a short time without delaying the progression of the cognitive disorders. Since 2003, no new drugs have been approved by the US Food and Drug Administration for the treatment of AD. In 2019, phase III clinical trials of crenezumab and aducanumab, two anti-amyloid beta monoclonal antibodies, were terminated. The scant progress in clinical trials urges efforts towards an alternative therapeutic strategy for dementia.

Approximately half of dementia patients suffered from depression. Physicians tend to prescribe selective serotonin reuptake inhibitors (SSRIs) for depression-complicating dementia. SSRIs have effects on brain function associated with neuronal plasticity, neurogenesis, and neuronal differentiation in rodents and may contribute to cognitive performance. However, the role of SSRIs in the cognitive function of dementia patients has not yet reached a consensus. Therefore, we conducted the present meta-analysis to review and quantify the effects of SSRIs, especially fluoxetine, on cognitive function in AD and VaD patients, with the intention to provide useful advice for clinical practice.

The literature search initially identified 854 citations, and 14 randomized controlled trials (RCTs) published between 2000 and 2018 remained in the final analysis (Supplementary Fig. 1), of which five compared fluoxetine with a placebo.^1–5^ A total of 1091 patients (550 received SSRIs and 541 received the placebo) were followed from 8 to 12 weeks. The baseline information and quality assessment results of the included studies are presented in Supplementary Table 1 and Supplementary Fig. 2. The mean difference (MD) with 95% confidence intervals (CIs) was used as the effect size. No significant heterogeneity was detected for age, and there was no age difference (Fig. 1a) between the SSRI group and the control group.

**Fig. 1:**
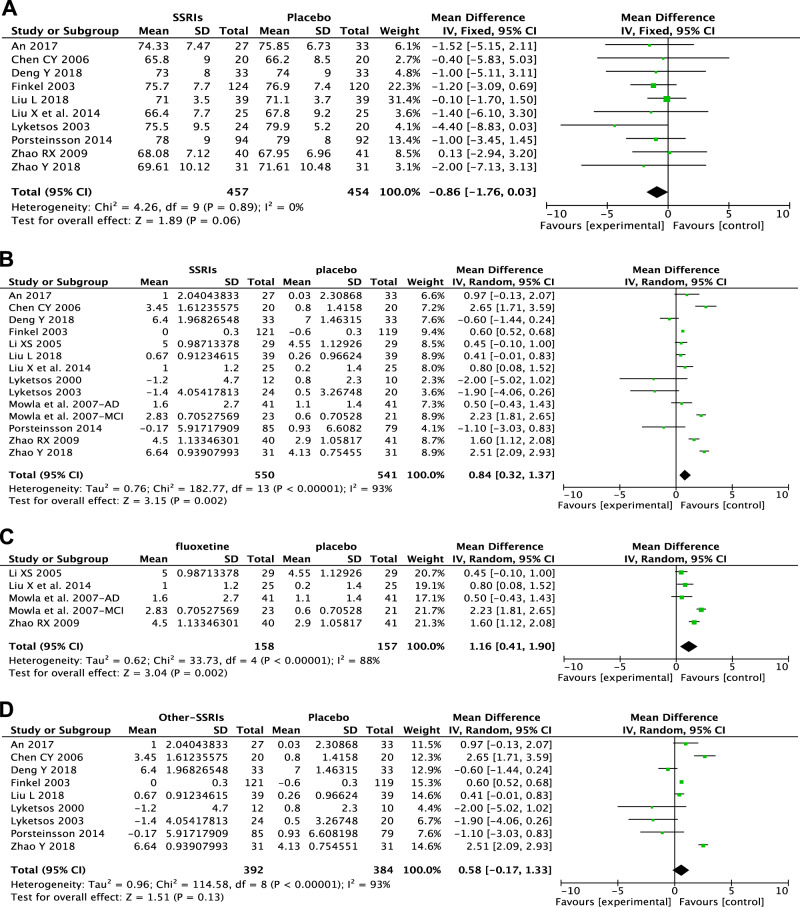
Forest plot of comparisons. **a** Forest plot of age comparison; there is no age difference between the SSRI and placebo groups (MD = −0.86, 95% CI: −1.76–0.03, *P* = 0.06). No significant heterogeneity existed (*I*^*2*^ = 0%, *P* = 0.89) **b** Forest plot of overall MMSE; there was a significant difference between the SSRI and placebo groups (MD = 0.84, 95% CI: 0.32–1.37, *P* = 0.002). **c** Fluoxetine significantly improved cognitive function in clinical practice (MD = 1.16, 95% CI: 0.41–1.90, *P* = 0.002). Significant heterogeneity existed (*I*^*2*^ = 88%, *P* < 0.00001). **d** There was no significant difference between nonfluoxetine SSRIs versus the placebo (MD = 0.58, 95% CI: −0.17–1.33, *P* = 0.13)

The effect of SSRIs on cognitive function as assessed by the Mini-Mental State Examination (MMSE) was the primary outcome of interest in this study. The MMSE scores differed between the SSRI and placebo groups, indicating a significantly beneficial influence of SSRIs on cognitive disorders compared with placebo (MD = 0.84, 95% CI: 0.32–1.37, *P* = 0.002) (Fig. 1b).

Two subgroups (fluoxetine and nonfluoxetine) of SSRIs were further analyzed. Five studies provided data for the fluoxetine subgroup comparison, showing a statistically significant difference in the MMSE scores between the fluoxetine and placebo groups (MD = 1.16, 95% CI: 0.41–1.90, *P* = 0.002) (Fig. 1c). Nine studies showed no significant difference between the nonfluoxetine SSRI and placebo groups with regard to the MMSE scores (MD = 0.58, 95% CI: −0.17–1.33, *P* = 0.13) (Fig. 1d).

The present meta-analysis explored the effectiveness of SSRIs with regard to the amelioration of cognitive performance in people with AD and VaD. All participants had a standardized diagnosis of dementia that was restricted to AD and VaD. Some, but not all, studies required participants to have depression at the point of entry. Our pooled meta-analysis is the first to support that SSRIs (mainly fluoxetine) exert beneficial effects on cognition compared with a placebo.

Previously, three meta-analyses investigated whether SSRIs influence cognitive performance in dementia patients and showed either a disadvantage or no benefit in the SSRI group.^6–8^ Previous studies recruited both AD and frontotemporal dementia patients and calculated the overall effects of SSRIs. Here, we focused on the effect of fluoxetine on AD and VaD, two of the most common types of dementia. Based on these meta-analyses, short-term SSRI treatment (<30 days) was significantly associated with increased risks of dementia compared with the placebo.^8^ Another study found increased rates of dementia during the initial prescription periods and then reductions in the rate of dementia during continued long-term antidepressant treatment.^9^ However, our meta-analysis demonstrates that fluoxetine treatment for 8–12 weeks is associated with improved MMSE performance (Fig. 1c). Interestingly, this conclusion is consistent with and supported by the fact that fluoxetine can trigger neurogenesis and synapse formation in the rat hippocampus and enlarge hippocampal volume in female patients; more detailed potential mechanisms are discussed in our accompanying review.^10^ As depression impairs cognitive function, improved cognition might be expected after the remission of depression due to SSRI treatment; however, this is unlikely to be the underlying mechanism because only fluoxetine and no other SSRIs exerted a beneficial influence on cognition compared with the placebo in our meta-analysis.

Our study had several strengths. To minimize variations in types of cognition tests, we only calculated the MDs of the MMSE and avoided mixing different types of tests together. With the available evidence and the enlarged number of RCTs studies to date, we have enhanced statistical power to quantify the effects of SSRI antidepressants on cognition in dementia patients. In addition, the quality of the included studies was generally high, and subgroup analyses were carried out to reduce heterogeneity. The sensitivity analysis showed that the estimates were relatively stable. Limitations should be considered. Although strict inclusion criteria and random effects models were applied, significant heterogeneity still existed. Only 14 studies met the eligibility criteria, and fewer were eligible for the subgroup analysis with a short follow-up period, which could limit the strength of the conclusions.

In light of the growing evidence from recent RCTs, our systematic review revealed for the first time an important alleviating effect of SSRIs, mainly fluoxetine, on cognitive performance in AD and VaD patients. Finally, we propose longer follow-up (1 year) and multicenter RCTs, which also employ objective and reliable imaging examinations (such as the volume ratio of the lateral ventricle to the hippocampus as a standard evaluation).^10^ Studying a large number of patients with fluoxetine treatment for 1 year may elucidate its ultimate efficacy.

## Supplementary information

Supplementary data.

## References

[1] Li XS (2005). The curative effects of fluoxetine on vascular dementia. J. Linyi Med. Coll..

[2] Mowla A, Mosavinasab M, Haghshenas H, Haghighi AB (2007). Does serotonin augmentation have any effect on cognition and activities of daily living in Alzheimer’s dementia? A double-blind, placebo-controlled clinical trial. J. Clin. Psychopharmacol..

[3] Mowla A, Mosavinasab M, Pani A (2007). Does fluoxetine have any effect on the cognition of patients with mild cognitive impairment? A double-blind, placebo-controlled, clinical trial. J. Clin. Psychopharmacol..

[4] Zhao RX, Ren AH (2009). Effect of different doses of fluoxetine in the treatment of vascular dementia. J. Huaihai Med..

[5] Liu X (2014). Effects of fluoxetine on brain-derived neurotrophic factor serum concentration and cognition in patients with vascular dementia. Clin. Interv. Aging.

[6] Sepehry AA, Lee PE, Hsiung GY, Beattie BL, Jacova C (2012). Effect of selective serotonin reuptake inhibitors in Alzheimer’s disease with comorbid depression: a meta-analysis of depression and cognitive outcomes. Drugs Aging.

[7] Jones HE, Joshi A, Shenkin S, Mead GE (2016). The effect of treatment with selective serotonin reuptake inhibitors in comparison to placebo in the progression of dementia: a systematic review and meta-analysis. Age Ageing.

[8] Wang YC (2018). Increased risk of dementia in patients with antidepressants: a meta-analysis of observational studies. Behav. Neurol..

[9] Kessing LV, Sondergard L, Forman JL, Andersen PK (2009). Antidepressants and dementia. J. Affect. Disord..

[10] Liu, P.-P., Xie, Y. & Kang, J.-S. History and progress of hypotheses and clinical trials for Alzheimer’s disease. *Signal Transduct. Target. Ther.*10.1038/s41392-019-0063-8 (2019). 10.1038/s41392-019-0063-8 PMC6799833 31637009

