# Supplementary material for: The effect of selective serotonin reuptake inhibitors on cognitive function in patients with Alzheimer’s disease and vascular dementia: focusing on fluoxetine with long follow-up periods
Source: Signal Transduct Target Ther. 2019 Aug 30;4:30. doi: 10.1038/s41392-019-0064-7 (PMC6799811; doi:10.1038/s41392-019-0064-7)
Supplement: Supplementary file 1 — Supplementary data. [file 41392_2019_64_MOESM1_ESM.pdf]

The effect of selective serotonin reuptake inhibitors on cognitive function of Alzheimer's disease and Vascular Dementia: focusing on fluoxetine with long follow-up period

Yi Xie[1,2], Pei-Pei Liu[1], Ya-Jun Lian[2], Hong-Bo Liu[2], Jian-Sheng Kang[1]

1. Clinical Systems Biology Laboratories, The first affiliated Hospital of Zhengzhou University, Zhengzhou, 450052, Henan, China
2. Department of Neurology, The first affiliated Hospital of Zhengzhou University, Zhengzhou, 450052, Henan, China

Correspondence: Jian-Sheng Kang, E-mail: kjs@zzu.edu.cn ORCID ID 0000-0002-2603-9718.

Ya-Jun Lian E-mail: E-mail: xysjkx@hotmail.com

Hong-Bo Liu E-mail: E-mail: fccliuhb@zzu.edu.cn

Keywords: Alzheimer's disease; Vascular dementia; SSRIs; Fluoxetine.

Methods

Search strategy

We systematically searched Pubmed, Embase and Cochrane Library for all relevant English languages articles and WanFang, Vip, and Chinese Knowledge Resource Integrate (CNKI) for all relevant Chinese languages articles published up to January 2019. Medical subject heading (MeSH) terms and text words including SSRIs, fluoxetine, escitalopram, citalopram, paroxetine, fluvoxamine, sertraline, dementia, cognition disorders were entered. We searched all relevant articles based on the consensus among authors and manually explored their reference lists for potential articles.

Inclusion and exclusion criteria

Inclusion criteria are as follows: (1) RCTs that the enrolled patients diagnosed as AD or VaD or mild cognitive impairment (MCI). (2) patients were divided into SSRIs group (SSRIs group refers to the participants treated with any type of SSRIs) or control group (control group refers to participants treated with placebo). (3) pre- and post- treatment cognitive data obtained through at least one standardized instrument. (4) study results adequately reported in the form of mean and standard deviation. (5) follow-up lasted no less than 8 weeks.

Exclusion criteria are as follows: (1) retrospective, preclinical studies, reviews, and meta-analysis not containing primary data. (2) duplicate reports of the same population.

Study selection and data extraction

Two authors (Y.X. and P-P.L.) independently checked the titles and abstracts of studies that potentially met the inclusion criteria and resolved any uncertainty through discussion. Data were extracted independently by two reviewers (Y.X. and P-P.L.) and entered onto a standardized, pre-piloted data extraction form for assessment of study quality and evidence synthesis. The extracted characteristics were data on age, sex, antidepressant dosage, follow-up period, case number and MMSE results. If missing data were presented in an original study for certain outcomes, the method of imputations was used.

Quality assessment

Two reviewers (H-B.L and Y-J.L) evaluated the quality of each selected study using the Cochrane Collaboration's tool for assessing risk and any disagreement was resolved by discussion. We assessed the quality of studies by reporting the following domains: random sequence generation, allocation concealment, binding of participants and personnel, binding of outcome assessment, incomplete outcome data, selective reporting and other bias, and each evaluated with "high risk of bias", "low risk of bias" and "unclear risk of bias"[1].

Statistical analysis

All analyses were performed using Review Manager 5.2. The I^2 statistic and Cochrane Q test were used to evaluate between-study heterogeneity. An $I^2 > 50\%$ or $P < 0.05$ indicated significant heterogeneity, and a random-effects model was used for meta-analysis; otherwise, a fixed-effects model was used[1]. The pre- and post-treatment mean difference (MD) and corresponding 95% confidence interval (95% CI) of MMSE scores were used as the effect

size for the pooled analysis. If not directly given in article, effect size was calculated by means recommended in Cochrane handbook version 5.1.0. A 95% CI without covering zero indicated a statistical significance of the pooled results. We did sensitivity analyses by changing effect models (fixed-effects model and random-effects model).

Table 1. basic information of included studies.

Study	Year	SSRIs	Diagnosis	SSRIs/ control	Dosage mg/d	Age Fluoxetine/control	Length of follow-up
Lyketsos et al.[2]	2000	sertraline	Probable AD, DSM- major depressive	12/10	50-150	-	9 weeks
Lyketsos et al.[3]	2003	sertraline	Probable AD, DSM- major depressive	24/20	50-150	75.5±9.5/79.9±5.2	12 weeks
Finkel et al.[4]	2004	sertraline	Probable or possible AD	124/120	25	75.7±7.7/76.9±7.4	8 weeks
Li XS et al.[5]	2005	fluoxetine	VaD	29/34	20	-	8 weeks
Chen CY et al.[6]	2006	citalopram	Post-stroke cognitive impairment	20/20	20	65.8±9.0/66.2±8.5	12 weeks
Mowla et al.[7]	2007	fluoxetine	mild to moderate AD	41/41	20	-	12 weeks
Mowla et al.[8]	2007	fluoxetine	MCI	23/21	20	-	8 weeks
Zhao RX et al.[9]	2009	fluoxetine	Probable VaD	40/41	20	68.08±7.1/67.95±6.96	12 weeks
Liu X et al.[10]	2014	fluoxetine	VaD	25/25	20	66.4±7.7/67.8±9.2	12 weeks
Porsteinsson et al.[11]	2014	citalopram	Probable AD	94/92	30	78±9/79±8	9 weeks
An et al.[12]	2017	escitalopram	AD, olin provisional criteria for depression	27/33	5 − 15	74.33±7.5/75.85±6.7	12 weeks
Deng Y et al.[13]	2018	sertraline, paroxetine, citalopram	AD, depression	33/33	-	73±8/74±9	12 weeks
Liu L et al.[14]	2018	citalopram	AD, at least one BPSD	39/39	10-30	71±3.5/71.1±3.7	12 weeks
Zhao Y et al.[15]	2018	paroxetine	VaD	31/31	10-20	69.61±10.1/71.61±10.5	12 weeks

Although the majority of studies (n = 8) included AD patients, four included VaD, one with post-stroke cognitive impairment and one mild cognitive impairment (MCI), and thus further analysis need to explore how dementia subtypes respond to SSRIs.

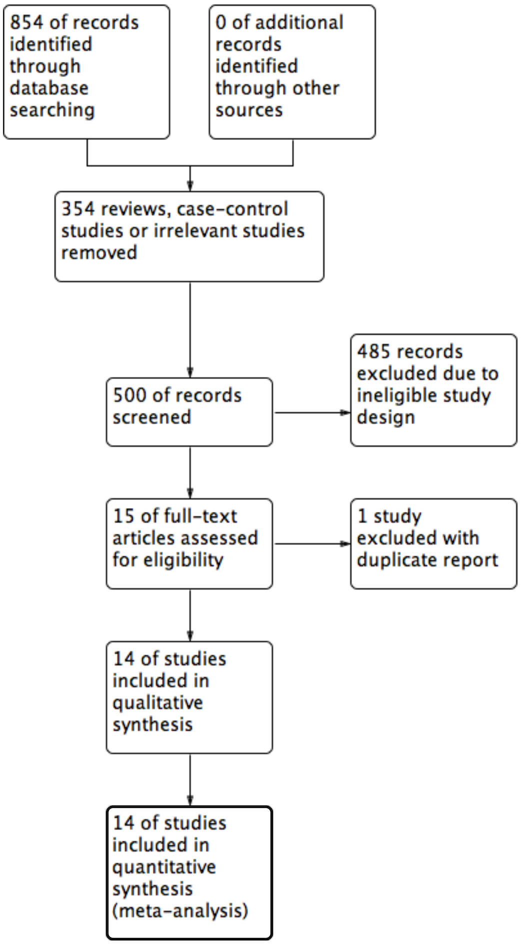

Figure 1. Flow chart of the meta-analysis

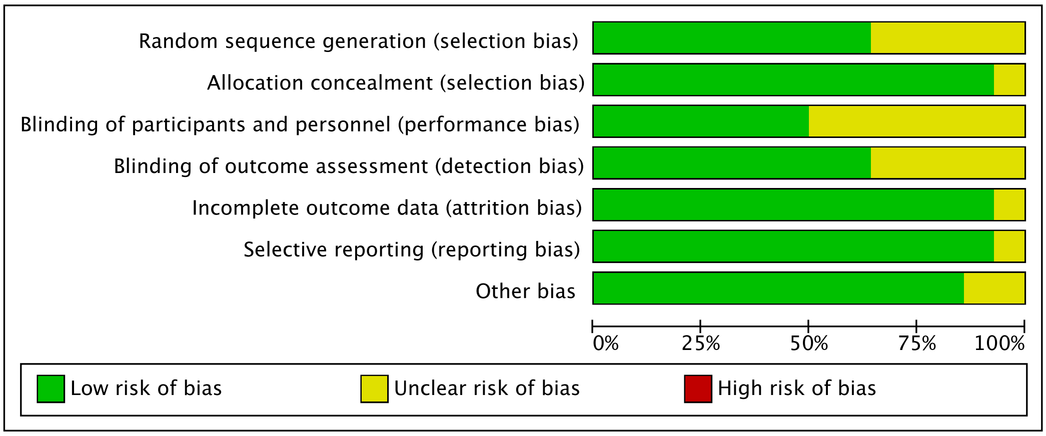

Figure 2. Risk of bias graph: judgements about each risk of bias item presented as percentages across all included studies

Reference

1. Higgins, J.P.T. & Green, S. Cochrane Handbook for Systematic Reviews of Interventions, Version 5.1.0. (The Cochrane Collaboration, 2011) http://handbook.cochrane.org/.
2. Lyketsos, C. G. et al. Randomized, placebo-controlled, double-blind clinical trial of sertraline in the treatment of depression complicating Alzheimer's disease: initial results from the Depression in Alzheimer's Disease study. *The American journal of psychiatry* **157**, 1686-1689, doi:10.1176/appi.ajp.157.10.1686 (2000).
3. Lyketsos, C. G. et al. Treating depression in Alzheimer disease: efficacy and safety of sertraline therapy, and the benefits of depression reduction: the DIADS. *Archives of general psychiatry* **60**, 737-746, doi:10.1001/archpsyc.60.7.737 (2003).
4. Finkel, S. I. et al. A randomized, placebo-controlled study of the efficacy and safety of sertraline in the treatment of the behavioral manifestations of Alzheimer's disease in outpatients treated with donepezil. *International journal of geriatric psychiatry* **19**, 9-18, doi:10.1002/gps.998 (2004).
5. Li, X.S. The curative effects of fluoxetine on vascular dementia. *Journal of Linyi Medical College* **27**, 435-437 (2005).
6. Chen, C.Y. Chen, K.N., Liu, C.Y., Duan, W. Influence of SSRIs(selective serotonin reuptake inhibitors)/citalopram on cognitive impairment in patients with stroke. *Journal of Chongqing Medical University* **31**, 240-242 (2006).
7. Mowla, A., Mosavinasab, M., Haghshenas, H. & Haghighi, A.B. Does serotonin augmentation have any effect on cognition and activities of daily living in Alzheimer's dementia? A double-blind, placebo-controlled clinical trial. *Journal of Clinical Psychopharmacology* **27**, 484-487 (2007).
8. Mowla, A., Mosavinasab, M. & Pani, A. Does fluoxetine have any effect on the cognition of patients with mild cognitive impairment? A double-blind, placebo-controlled, clinical trial. *Journal of clinical psychopharmacology* **27**, 67-70, doi:10.1097/JCP.0b013e31802e0002 (2007).
9. Zhao, R.X. & Ren, A.H. Effect of different doses of fluoxetine in the treatment of vascular dementia. *J. Huaihai Med.* **27**,425 (2009).
10. Liu, X. et al. Effects of fluoxetine on brain-derived neurotrophic factor serum concentration and cognition in patients with vascular dementia. *Clinical interventions in aging* **9**, 411-418, doi:10.2147/CIA.S58830 (2014).
11. Porsteinsson, A. P. et al. Effect of citalopram on agitation in Alzheimer disease: the CitAD randomized clinical trial. *Jama* **311**, 682-691, doi:10.1001/jama.2014.93 (2014).
12. An, H. et al. The Effect of Escitalopram on Mood and Cognition in Depressive Alzheimer's Disease Subjects. *Journal of Alzheimer's disease : JAD* **55**, 727-735, doi:10.3233/jad-160225 (2017).
13. Deng, Y., Lou, Y.J., Zhong, M.J. & Deng, L. Clinical study of memantine combined with antidepressants in the treatment of AD with depression and anxiety and agitation. *Medicine in practice* **8**, 48-51 (2018).
14. Liu, L., Kong, L.L., Wang, C.X. & Liu, C.W. Comparative study of citalopram in treatment of behavioral and psychological symptoms of dementia and cognitive

function in Alzheimer's disease patients. *Chinese Journal of Behavioral Medicine and Brain Science* **27**, 52-55 (2018).

15. Zhao, Y., Gu, S.G. & Wang, D.G. Effect of paroxetine on cognitive function in patients with mild to moderate vascular dementia. *China Journal of Health Psychology* **26**, 1508-1511 (2018).
